# Correction: Lee et al. Alleviation of Immobilization Stress or Fecal Microbiota-Induced Insomnia and Depression-like Behaviors in Mice by *Lactobacillus plantarum* and Its Supplement. *Nutrients* 2024, *16*, 3711

**DOI:** 10.3390/nu17081338

**Published:** 2025-04-14

**Authors:** Dong-Yun Lee, Ji-Su Baek, Yoon-Jung Shin, Dong-Hyun Kim

**Affiliations:** 1 Neurobiota Research Center, College of Pharmacy, Kyung Hee University, 26, Kyungheedae-ro, Dongdaemun-Gu, Seoul 02447, Republic of Korea; dongyun8246@naver.com (D.-Y.L.); bjisoo500@naver.com (J.-S.B.); nayo971111@naver.com (Y.-J.S.); 2 PBLbioLab, Inc., Seoul 03174, Republic of Korea

## Error in Figure

In the original publication [1], there was a mistake in Figure 3 as published. The corrected Figure 3 appears below. The authors state that the scientific conclusions are unaffected. This correction was approved by the Academic Editor. The original publication has also been updated.

## Figures and Tables

**Figure 3:**
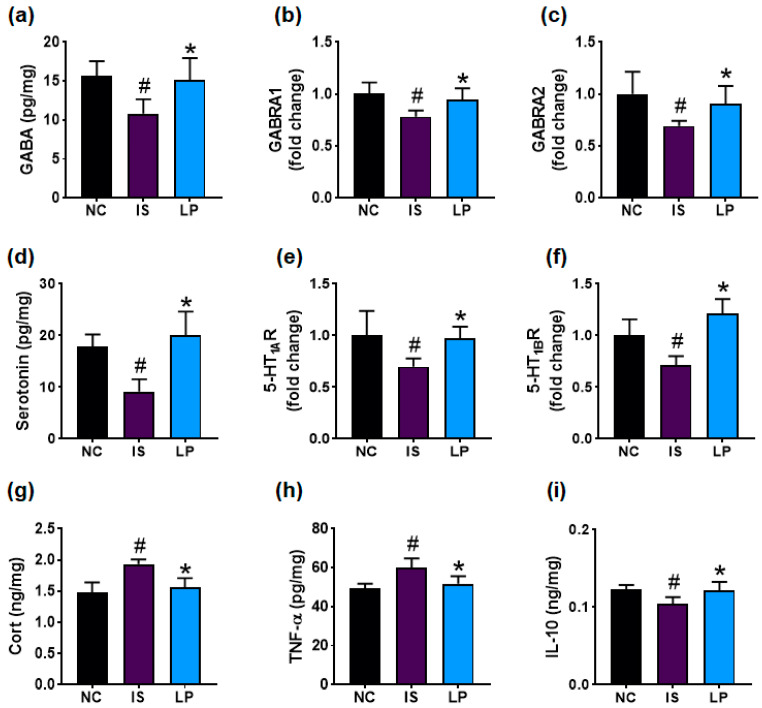
Effect of P72 on the expression of DA- and insomnia-related markers in the prefrontal cortex. Effect on GABA (**a**), GABA_A_Rα1 (**b**), and GABA_A_Rα2 (**c**) expression levels. Effect on serotonin (**d**), 5-HT_1A_R (**e**), and 5-HT_1B_R (**f**) expression levels. Effect on corticosterone (Cort, (**g**)), TNF-α (**h**), and IL-10 (**i**) levels. IS, vehicle; P72, 1 *×* 10^9^ CFU/mouse/day of P72 in IS/IF-treated mice; NC, saline in IS/IF-nontreated mice. Data are mean ± SD (n = 8). ^#^ *p* < 0.05 vs. NC. * *p* < 0.05 vs. IS.
