# Supplementary material for: Alleviation of Immobilization Stress or Fecal Microbiota-Induced Insomnia and Depression-like Behaviors in Mice by Lactobacillus plantarum and Its Supplement
Source: Nutrients. 2024 Oct 30;16(21):3711. doi: 10.3390/nu16213711 (PMC11547846; doi:10.3390/nu16213711)
Supplement: Supplementary file 1 [file nutrients-16-03711-s001.zip › nutrients-3208220-SI.pdf]

Alleviation of immobilization stress or fecal microbiota-induced insomnia and depression-like behaviors in mice by *Lactobacillus plantarum* and its supplement

Table S1. Primers used in this study

	Primer	
	Forward	Reverse
$GABA_A$ receptor α1	5′- GAGTCGTCCAATCCAGCAC-3′	5′- AGCCAGAAGGAAACCTGTGA-3′
$GABA_A$ receptor α2	5′- TTACAGTCCAAGCCGAATGTCCC-3′	5′- ACTTCTGAGGTTGTGTAAGCGTAGC-3′
MT1R	5′- TGTCAGCGAGCTGCTCAATG-3′	5′- GGTACACAGACAGGATGACCA-3′
MT2R	5′- GAACAGCTCAATCCCTAACTGC-3’	5′- ACGACTACTGTAGATAGCATGGG-3’
5-HT_{1A}R	5′- CCGTGAGAGGAAGACAGTCTAAGA-3′	5′- GGTTGAGCAGGGAGTTGGAGTAG-3′
5-HT_{1B}R	5′- CCAGCGGTCCATCCACAGAG-3′	5′- CCAGCGGTCCATCCACAGAG-3′
GAPDH	5′-TGCAGTGGCAAAGTGGAGAT-3′	5′-TTTGCCGTGAGTGGAGTCATA-3′

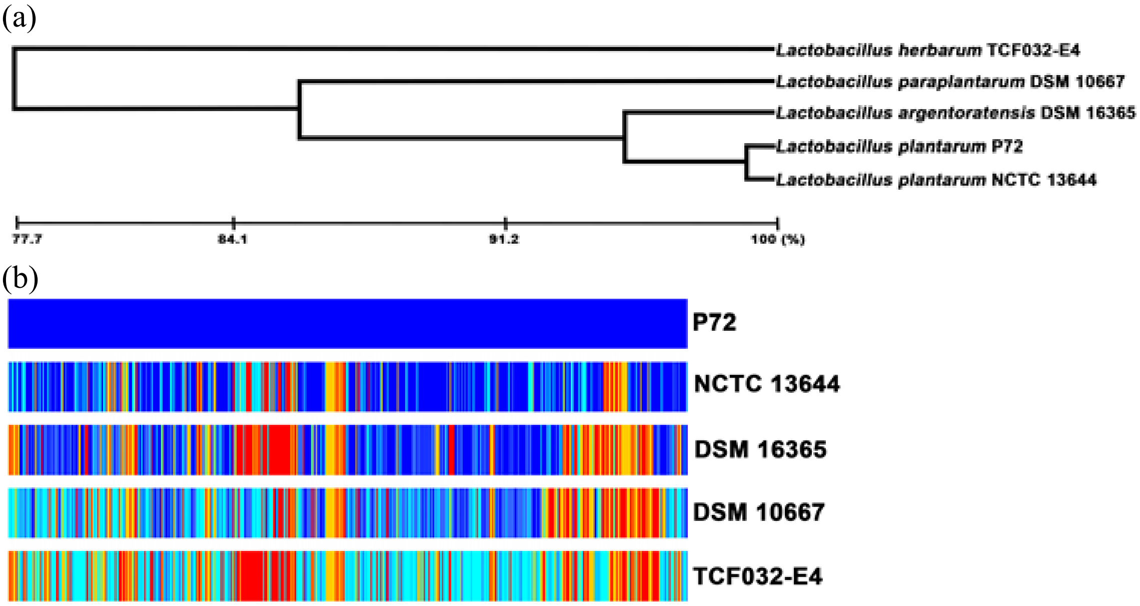

Figure S1. Taxonomic classification by genome-wide comparative analysis of P72. (a) Neighbor-joining tree based on the OrthoANI distance matrix (analyzed using UPGMA dendrogram, Newick format). (b) The pairwise ortholog matrix table (generated and colored according to the similarity between matching sequences).

Methods

Preparation of mice with immobilization stress

Immobilization stress (IS) was performed, as follows: each mouse was inserted into a conical tube-like instrument (2.5 cm in diameter, 7.5 cm in length) with a 0.25-cm-diameter hole on the center of the tube) and vertically placed for 8 h/day and repeated for 5 days, as previously reported [1].

Behavioral tasks

Depression- and sleepless-like behaviors were measured in a room equipped with a record camera and quantified using the EthoVision XT software. The OFT was performed in a chamber (40×40 cm; center zone, 20×20 cm) for 10 min [2]. The EPMT was measured in a

plus maze apparatus consisting of two open [30 × 7 cm] and two enclosed [30 × 7 cm] arm with 20-cm-high walls extending from a central platform [7 × 7 cm] [3]. The TST was measured on the edge of a table, at 30 cm above it [3].

Sleeping tests for sleep latency time (SLT) and sleep duration (SD) were measured under the exposure to pentobarbital sodium or isoflurane [4]. Pentobarbital sodium (40 mg/kg, Hanlim Pharm. Co., Ltd., Korea, diluted in sterilized saline) was intraperitoneally injected or isofurane (2%) in a chamber was exposed for 10 min from next day after the final depression-like behavioral task. Diphenhydramine (20 mg/kg) was orally gavaged 30 min before the injection of pentobarbital sodium.-The time of righting reflex loss and its appearance were measured for 10 and 60 min in isofurane- and pentobarbital-induced sleep test, respectively. Latency time for righting reflex (SLT) and SD (the time from righting reflex loss to righting reflex recovery) were measured.

Enzyme-linked immunosorbent assay (ELISA)

Collected brain (prefrontal cortx) and colon tissues were homogenized and lysed in RIPA buffer (150 mM sodium chloride, 1% sodium deoxycholate, 1% Triton X-100, 0.1% SDS, 50 mM Tris–HCl, 2 mM EDTA, pH 7.5) containing a phosphatase inhibitor cocktail (Roche) and centrifuged at 10,000 *g* and 4°C for 20 min, as previously reported [5].

In the supernatant, TNF-α, IL-1β, IL-6, IL-10, and myeloperoxidase (R&D system, Minneapolis, Mn), corticosterone (eBioscience, TX), GABA (Mybiosource, San Diego, CA), and serotonin (DLD Diagnositika GmbH, Hamburg, Germany) levels were measured using their ELISA kits.

Quantitative polymerase chain reaction (qPCR)

mRNAs (2 μg) from the prefrontal and thalamus were isolated using a RNeasy Mini kit and their cDNAs were prepared using cDNA synthesis kit (TaKaRa) [5]. The real time qPCR for serotonin 1A receptor (5-HT_{1A}R), 5-HT_{1B}R, GABA type A receptor subunit alpha1 ($GABA_A$Rα1), GABA type A receptor subunit alpha2 ($GABA_A$Rα2), melatonin receptor type 1 (MT1R), melatonin receptor type 2 (MT2R), and glyceraldehyde-3-phosphate dehydrogenase (GAPDH) was performed using SYBER premix Ex Taq II (TaKaRa). The thermal cycling condition was as follows: initial denaturation at 95°C for 30 s, denaturation at 95°C for 15 s, annealing at 60°C for 30 s, extension at 72°C for 30 s, and 40 cycling. Gene expression levels were calculated by comparing to GAPDH. Primers are shown in Supplement Table S1.

Immunofluorescence staining

The immunofluorescence staining of brain and colon tissues were performed according to the method of Lee et al. [6]. Briefly, mice were trans-cardiacally perfused with 4% paraformaldehyde and removed brains and colons. These tissues were post-fixed with 4% paraformaldehyde for 4 h, cytoprotected in 30% sucrose solution, freezed, and sectioned using a cryostat. The sections were incubated for 16 h at 4°C with primary antibodies for $GABA_A$Rα1, NF-κB, Iba1, and/or CD11c, washed with saline twice, and incubated with secondary antibodies conjugated with Alexa Fluor 488 (1:1,000, Invitrogen) or Alexa Fluor 594 (1:500, Invitrogen). The nuclei were stained with DAPI. Immunostained sections were observed with a confocal laser microscope.

References

1. Jang, H.M.; Lee, K.E.; Lee, H.J.; Kim, D.H. Immobilization stress-induced Escherichia coli causes anxiety by inducing NF-κB activation through gut microbiota

disturbance. *Sci Rep* **2018**, *8*, 13897, doi:10.1038/s41598-018-31764-0.
2. Joo, M.K.; Ma, X.; Yoo, J.W.; Shin, Y.J.; Kim, H.J.; Kim, D.H. Patient-derived Enterococcus mundtii and its capsular polysaccharides cause depression through the downregulation of NF-κB-involved serotonin and BDNF expression. *Microbes Infect* **2023**, 105116, doi:10.1016/j.micinf.2023.105116.
3. Jang, H.M.; Lee, K.E.; Kim, D.H. The Preventive and Curative Effects of Lactobacillus reuteri NK33 and Bifidobacterium adolescentis NK98 on Immobilization Stress-Induced Anxiety/Depression and Colitis in Mice. *Nutrients* **2019**, *11*, doi:10.3390/nu11040819.
4. Ma, Y.; Ma, H.; Eun, J.S.; Nam, S.Y.; Kim, Y.B.; Hong, J.T.; Lee, M.K.; Oh, K.W. Methanol extract of Longanae Arillus augments pentobarbital-induced sleep behaviors through the modification of GABAergic systems. *J Ethnopharmacol* **2009**, *122*, 245-250, doi:10.1016/j.jep.2009.01.012.
5. Joo, M.K.; Ma, X.; Yoo, J.W.; Shin, Y.J.; Kim, H.J.; Kim, D.H. Patient-derived Enterococcus mundtii and its capsular polysaccharides cause depression through the downregulation of NF-κB-involved serotonin and BDNF expression. *Microbes Infect* **2023**, *25*, 105116, doi:10.1016/j.micinf.2023.105116.
6. Lee, K.E.; Kim, J.K.; Han, S.K.; Lee, D.Y.; Lee, H.J.; Yim, S.V.; Kim, D.H. The extracellular vesicle of gut microbial Paenalcaligenes hominis is a risk factor for vagus nerve-mediated cognitive impairment. *Microbiome* **2020**, *8*, 107, doi:10.1186/s40168-020-00881-2.
